# Digitale Gesundheitsanwendungen aus regulatorischer Sicht – Erfahrungen und aktuelle Entwicklungen

**DOI:** 10.1007/s00115-025-01878-8

**Published:** 2025-08-18

**Authors:** Florian Strauch, Karl Broich

**Affiliations:** Bundesinstitut für Arzneimittel und Medizinprodukte (BfArM), Kurt-Georg-Kiesinger-Allee 3, 53175 Bonn, Deutschland

**Keywords:** Psychiatrische Indikationen, Fast Track, DiGA-Verzeichnis, DiGA-Leitfaden, Digital-Gesetz, Psychiatric indications, Fast track, DiGA directory, DiGA guide, Digital Act

## Abstract

Seit 2020 können digitale Gesundheitsanwendungen, kurz DiGA, auf Rezept verordnet und von den Krankenkassen erstattet werden und sind damit in der Patientenversorgung zu einem der Vorreiter in der Integration digitaler Therapeutika geworden. Voraussetzung für diese „Apps auf Rezept“ ist die Listung im DiGA-Verzeichnis nach positiv durchlaufenem Bewertungsverfahren beim Bundesinstitut für Arzneimittel und Medizinprodukte (BfArM). Im DiGA-Verzeichnis des BfArM finden sich adressatengerecht und transparent aufbereitete Informationen zu den einzelnen DiGAs und deren Eigenschaften. Neben einem positiven Versorgungseffekt muss eine DiGA umfangreiche Qualitäts‑, Datenschutz- und Sicherheitsparameter erfüllen. 28 von den 59 derzeit verschreibungsfähigen DiGAs befassen sich mit psychiatrischen Indikationen. Der Nachweis der positiven Versorgungseffekte wurde ganz überwiegend in randomisierten kontrollierten Studien gezeigt. DiGAs sollten nach einer sachgerechten Diagnostik, Indikationsstellung und Aufklärung komplementär, d. h. therapievorbereitend und therapiebegleitend, eingesetzt werden. Eine Verordnung ist durch ärztliche oder psychotherapeutische Praxen, aber auch aus der stationären Behandlung über das Entlassmanagement möglich. Patientinnen und Patienten, die DiGAs einsetzen, schätzen deren Verfügbarkeit in ihrem individuellen Alltag, die Informations- und Austauschmöglichkeiten im Rahmen des Therapiesettings und das gestärkte Selbstmanagement.

**Zusatzmaterial online:**

Die Online-Version dieses Beitrags (10.1007/s00115-025-01878-8) enthält eine Übersicht über die Begriffsdefinitionen.

## Abstract (en)

Since 2020 digital health applications, or DiGA for short, can be prescribed and reimbursed by health insurance companies, making them one of the pioneers in the integration of digital therapeutics in patient care. The prerequisite for these “apps on prescription” is a listing in the DiGA directory after successfully passing an evaluation process at the Federal Institute for Drugs and Medical Devices (BfArM). The DiGA directory of the BfArM contains information on the individual DiGAs and their properties that are transparent and tailored to the target group. In addition to a positive effect on care a DiGA must meet extensive quality, data protection and security parameters. Of the 59 DiGAs currently available on prescription 28 deal with psychiatric indications. The proof of positive effects on care were overwhelmingly demonstrated in randomized controlled trials. A DiGA should be used after proper diagnostics, determination of indications and education, both as a preparation for therapy and as a supplement to therapy. They can be prescribed by medical or psychotherapeutic practices but also by inpatient treatment providers as part of discharge management. Patients who use DiGAs appreciate their availability in their individual everyday lives, the opportunities for information and exchange within the therapy setting and the increased sense of self-management.

## Hintergrund

Das Digitale-Versorgung-Gesetz (DVG) führte 2019 digitale Gesundheitsanwendungen (DiGAs) als Leistung der gesetzlichen Krankenversicherung ein, die DiGAs waren damit ein Vorreiter bei der Einführung digitaler Therapeutika in die Gesundheitsversorgung [7, 16]. Insgesamt 59 solcher Smartphone-Apps oder browserbasierter Webanwendungen wurden seitdem in das DiGA-Verzeichnis des Bundesinstituts für Arzneimittel und Medizinprodukte (BfArM) aufgenommen und ergänzen somit die Leistungen gesetzlich Versicherter bei unterschiedlichen Erkrankungen wie Diabetes mellitus, Rückenschmerzen oder Depressionen.

In der Digitale-Gesundheitsanwendungen-Verordnung (DiGAV) ist näher geregelt, welche Anforderungen DiGAs erfüllen müssen, um im DiGA-Verzeichnis gelistet zu werden. Formale Anforderungen reichen von Sicherheit und Funktionstauglichkeit (§ 3 DiGAV) über Datenschutz und Datensicherheit (§ 4 DiGAV) bis hin zu Anforderungen an die Qualität (§§ 5–6 DiGAV). Anforderungen an die Qualität sind insbesondere Interoperabilität, Werbe- und Barrierefreiheit. Der Nachweis positiver Versorgungseffekte (§§ 8–15 DiGAV) stellt den umfangreichsten Bereich der Prüfung durch das BfArM dar [6, 14].

In den letzten Jahren hat die Verordnung von DIGAs kontinuierlich zugenommen, so wurden zwischen den Jahren 2020 bis 2024 insgesamt ca. 861.000 DiGAs verordnet, wovon etwa 259.000 auf den Bereich der psychischen Erkrankungen fielen. Dabei ist es über die Jahre zu einem kontinuierlichen Anstieg gekommen: Im Vergleich zum Jahr 2023 verzeichnete die Inanspruchnahme von DiGAs im Jahr 2024 einen Anstieg von 85 %. Dabei wurden DiGAs zu 87 % überwiegend ärztlich oder psychotherapeutisch verordnet, der Anteil direkt eingelöster Freischaltcodes auf Basis einer dokumentierten Diagnose und entsprechender Genehmigung durch die Krankenkasse war mit 13 % eher gering [12].

Der vorliegende Artikel soll einen allgemeinen Überblick über relevante Eigenschaften und Entwicklungen seit dem Beginn des DiGA-Fast-Track-Verfahrens geben sowie absehbare Weiterentwicklungen und mögliche Potenziale aufzeigen. Dabei wird ein Schwerpunkt auf die Behandlung psychischer Erkrankungen gelegt. Zudem soll er Ärztinnen und Ärzten, die bisher keinen Kontakt mit DiGAs hatten, grundlegendes Wissen vermitteln und hilfreiche Anregungen bieten.

## Voraussetzungen und Eigenschaften einer DiGA

Digitale Gesundheitsanwendungen sind Medizinprodukte niedriger Risikoklassen (I oder IIa), d. h. viele grundsätzliche Anforderungen an Sicherheit und Funktionstauglichkeit werden in der Regel bereits dadurch erfüllt, dass jede DiGA ein CE-gekennzeichnetes Medizinprodukt nach Verordnung (EU) 2017/745 (MDR) sein muss. Im Rahmen einer Konformitätsbewertung müssen danach grundlegende Sicherheits- und Leistungsanforderungen erfüllt werden. Hersteller selbst führen zur Beurteilung dieser Anforderungen unter anderem eine erste klinische Bewertung durch, erstellen eine technische Dokumentation und verlangen ein Qualitätsmanagementsystem sowie ein Risikomanagementsystem unter Berücksichtigung relevanter Normen. Im Falle der niedrigen Risikoklasse I führt ein Hersteller diese Konformitätsbewertung selbst durch und dokumentiert diese, im Falle der höheren Risikoklassen IIa und IIb werden relevante Bereiche der Konformitätsbewertung des Herstellers von einer sog. Benannten Stelle (z. B. TÜV oder DEKRA) überprüft.

Erst nach dieser CE-Kennzeichnung und dem Inverkehrbringung des Medizinprodukts, dem Nachweis eines Informationssicherheitsmanagementsystems, dem Nachweis durchgeführter Datensicherheitstests sowie weiterer formaler Anforderungen werden Anträge auf Aufnahme in das DiGA-Verzeichnis seitens des BfArM überhaupt erst inhaltlich weiter geprüft. Dies erfolgt dann im „Fast-Track-Verfahren“ damit möglichst viele Patienten zeitnah von dieser neuen Versorgungsform profitieren.

Digitale Gesundheitsanwendungen müssen in der Regel die aktuelle Krankheitssituation der Patientinnen und Patienten anhand entsprechender Eingaben erfassen können und daraus krankheitsrelevante, auf die Patientinnen und Patienten zugeschnittene und operationalisierbare Rückmeldungen ausgeben. Eine hohe Individualisierbarkeit und Interaktionsmöglichkeiten sollen die Krankheit der Patientinnen und Patienten ins Zentrum rücken, auf deren individuelle, momentane Situation eingehen und sie zum selbstbestimmten Umgang mit ihrer Erkrankung befähigen. Zur Erfüllung der Eigenschaft einer DiGA reicht eine reine Zurverfügungstellung frei anwählbarer Funktionen, wie Symptomtagebücher, Gebrauchsinformationen für zusätzliche Hardware und Medikamente, Wissensmodule und Erinnerungsfunktionen, in der Regel nicht aus, wenn diese nicht miteinander verknüpft werden.

Jede DiGA muss einen menschen- und maschinenlesbaren Export relevanter Nutzungsdaten aus der Anwendung heraus ermöglichen. Somit können Patientinnen und Patienten jederzeit ihre Daten in einem interoperablen Format verwenden und beispielsweise zur Sprechstunde mitbringen.

Das BfArM prüft konkrete Anforderungen an Datenschutz und Datensicherheit im Rahmen des Antragsprozesses anhand von App-Zugängen und fordert Hersteller bei unzulässigen Abweichungen der Vorgaben der Anlage 1 DiGAV zu entsprechenden Änderungen auf. In naher Zukunft müssen DiGA-Hersteller stattdessen ein separates Zertifikat zum Nachweis der Erfüllung spezieller Anforderungen an die Datensicherheit einerseits und spezieller Anforderungen an den Datenschutz andererseits vorlegen. Die Anforderungen wurden in Zusammenarbeit mit dem Bundesamt für Sicherheit in der Informationstechnik (BSI) und der Bundesbeauftragten für Datenschutz und Informationsfreiheit (BfDI) entwickelt und werden zukünftig von akkreditierten Prüfstellen überprüft [4, 8].

Welche Nachweise müssen die Antragsteller über diese Anforderungen hinaus erbringen, um im DiGA-Verzeichnis des BfArM gelistet zu werden? Der Gesetzgeber hat dies in der Gesetzeskommentierung des DVG folgendermaßen formuliert:

„Der praktische Mehrwert durch die Gewinnung und Auswertung gesundheitsbezogener Daten, das geringe Risikopotenzial und die vergleichsweise niedrigen Kosten digitaler Gesundheitsanwendungen rechtfertigt es, für den Nachweis positiver Versorgungseffekte keine vergleichbar hohen Evidenzanforderungen zu stellen wie sie für den Nachweis des Zusatznutzens von Arzneimitteln mit neuen Wirkstoffen nach § 35a gefordert werden, die regelmäßig nur im Rahmen klinischer Studien höherer Evidenzstufe erbracht werden können, deren Aufwand hier jedoch unverhältnismäßig wäre.“

Gleichzeitig enthält der Gesetzesbeschluss die allgemeine Klarstellung, dass die Regelungen der DiGAV zum Nachweis positiver Versorgungseffekte „unter Berücksichtigung der Grundsätze evidenzbasierter Medizin“ erfolgt [7].

Das BfArM arbeitet daher in einem Spannungsfeld, in dem diese niedrigeren Evidenzanforderungen an DiGAs auf der einen Seite immer wieder kritisiert werden [12, 15, 17], auf der anderen Seite Antragsteller vorbringen, dass das BfArM in den letzten Jahren immer höhere Anforderungen stelle. Vom Gesetzgeber werden die geringeren Anforderungen im Rahmen des niedrigen Risikopotenzials gesehen, da es sich um Medizinprodukte der Risikoklassen I und IIa handelt. Konsequenterweise gilt das nicht mehr für die kommenden DiGAs, die in der Risikoklasse IIb eingeordnet sind, diese können nicht mehr vorläufig aufgenommen werden, sondern müssen direkt umfassendere Daten mit Evidenz für eine dauerhafte Listung erbringen [9]. Mit der Erweiterung auf Medizinprodukte der Klasse IIb werden umfassendere und wirkungsvollere DiGAs erwartet, die z. B. bei Patienten mit Diabetes mellitus oder anderen chronischen Erkrankungen auch Optionen mit Telemonitoring oder Fernüberwachung ermöglichen und in digitalen Disease-Management-Programmen gemäß § 137f des Fünften Buchs Sozialgesetzbuch (SGB V) integriert werden können [3, 7].

**Abb. 1:**
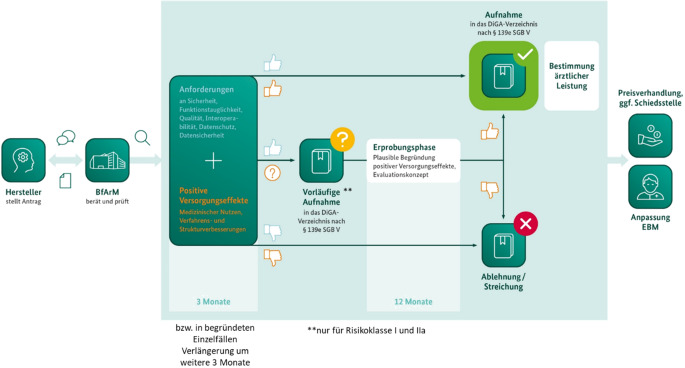
Fast-Track-Prozess am Bundesinstitut für Arzneimittel und Medizinprodukte (*BfArM*) zur Aufnahme einer digitalen Gesundheitsanwendung (*DiGA*) in das DiGA-Verzeichnis. *EBM* einheitlicher Bewertungsmaßstab, *SGB* Sozialgesetzbuch

**Abb. 2:**
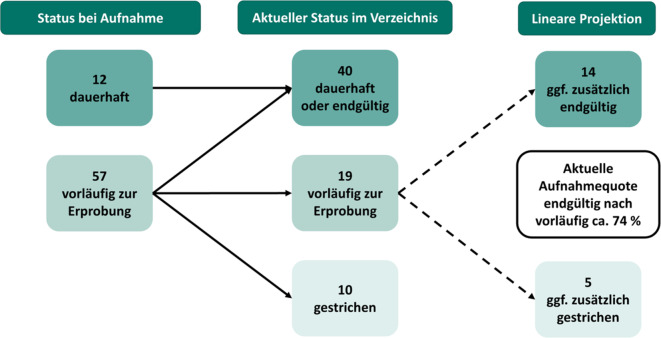
Aufnahmequote und Status der Antragstypen vorläufig und dauerhaft oder endgültig

Diesem zunächst niederschwelligen Ansatz folgend war es bewusst intendiert, dass DiGA-Hersteller zwischen zwei Arten von Anträgen auf Aufnahme in das DiGA-Verzeichnis wählen können (Abb. 1). Bei einem Antrag auf dauerhafte Aufnahme wird seitens des BfArM ein vollständiger Studienbericht einer bereits abgeschlossenen Studie mit ausreichender Evidenz vorausgesetzt. Dazu ist im Regelfall ein statistisch signifikanter und klinisch relevanter Unterschied im Vergleich zur Nichtnutzung der DiGA darzulegen. Bei einem Antrag auf vorläufige Aufnahme muss der Hersteller im Rahmen einer systematischen Datenauswertung ausreichend plausibel begründen, dass eine weitere Datenerhebung zur Bestätigung der bisher vorliegenden Daten zum Nachweis positiver Versorgungseffekte wahrscheinlich innerhalb eines Jahres erfolgreich sein wird, bei Bedarf kann dieser Zeitraum bis zu längstens 24 Monaten verlängert werden. In diesem Fall prüft das BfArM einerseits die plausible Begründung der Versorgungsverbesserung anhand erster Daten, welche in der Regel einarmige oder gar bereits randomisierte kontrollierte Vorstudien bzw. Interimsanalysen laufender Studien sein können [5, 16, 17, 19, 21]. Sobald der Erprobungszeitraum abgelaufen ist, trifft das BfArM anhand des vorzulegenden Studienberichts eine Entscheidung über die endgültige Aufnahme. In ca. 74 % der Fälle mündete eine vorläufige Aufnahme in einer endgültigen Aufnahme in das DiGA-Verzeichnis (Abb. 2). Ob eine DiGA vorläufig oder dauerhaft aufgenommen wurde, wird im DiGA-Verzeichnis transparent gemacht.

## Das Digitalgesetz – aktuelle, normative Entwicklungen

Das in 2024 in Kraft getretene Digitalgesetz (DigiG) hat umfangreiche Änderungen im Gesundheitswesen allgemein und im Speziellen für digitale Gesundheitsanwendungen gebracht [9]. Mit der neuen Möglichkeit der elektronischen Patientenakte (ePA) können Gesundheitsdaten zentral gespeichert und verwaltet werden. DiGA-Daten, die auch in die ePA geschrieben werden können, sollen tiefer in Versorgungsprozessen verankert werden. Um z. B. Technologien zur diagnostischen Fernüberwachung (Telemonitoring) von Risikopatienten zu ermöglichen, wurden die bisher geltenden Beschränkungen für DiGAs auf Medizinprodukte der Risikoklasse IIb erweitert. Weiter sollen alle im DiGA-Verzeichnis gelisteten DiGAs ab 2026 durch eine sog. anwendungsbegleitende Erfolgsmessung (AbEM) dauerhaft evaluiert werden. Das Gesetz formuliert dabei konkrete Erfolgskriterien der AbEM in § 139e Absatz 13 SGB V mit

1. der Dauer und Häufigkeit der Nutzung der DiGA,
2. der Patientenzufriedenheit mit der DiGA und
3. dem berichteten Gesundheitszustand während der DiGA-Nutzung [9].

Damit sollen einige der Hauptkritikpunkte der Leistungserbringer und der Kostenträger adressiert werden, der damit verbundene bürokratische Aufwand wird aber kritisch gesehen.

## Methodische Anforderungen an den Nutzennachweis

**Abb. 3:**
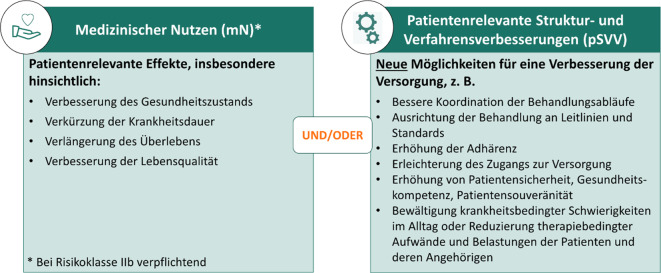
Mögliche Arten und Beispiele von positiven Versorgungseffekten bei digitalen Gesundheitsanwendungen

Was sind nun aber die positiven Versorgungseffekte, die von einer DiGA in klinischen Studien gezeigt werden müssen? Dies ist grundsätzlich in Form eines medizinischen Nutzens oder einer patientenrelevanten Struktur- und Verfahrensverbesserung (pSVV) möglich wie in Verordnung und BfArM-Leitfaden dargelegt (Abb. 3; [5, 6]).

Neben klassischen Endpunkten zur Messung der Krankheitssymptomatik oder Lebensqualität der DiGA-Anwendenden bieten pSVV neue Möglichkeiten für einen Nutzennachweis. Diese stellen eine einzigartige, neue Kategorie eines Versorgungsnutzens dar, der DiGAs insbesondere von klassischen Therapien (wie etwa die eines Arzneimittels) abhebt. Hierbei ist jedoch zu beachten, dass diese oftmals anhand mehrerer Studienendpunkte in den Kontext des Krankheitsverlaufs gesetzt werden müssen. So soll beispielweise vermieden werden, dass eine höhere Adhärenz zur Begleittherapie oder niedrigere Anzahl an Arztkontakten mit einer Verschlechterung des Gesundheitszustands einhergeht. Bislang stehen für diese Art des Nutzennachweises nicht immer validierte und etablierte Messinstrumente zur Verfügung. Zudem sind mögliche ökonomische Vorteile, die mit diesen Struktur- und Verfahrensverbesserungen einhergehen, nicht Teil der Bewertung des BfArM. Potenziale und Herausforderungen von pSVV wurden ausführlich in [10] diskutiert.

Der Leitfaden erläutert an vielen Beispielen die Voraussetzungen und Umsetzungsmöglichkeiten zur Entwicklung einer DiGA, zusätzlich empfiehlt das BfArM sehr die Nutzung seines wissenschaftlichen Beratungsangebots vor Festlegung des finalen Entwicklungsprogramms und des Studiendesigns. Ganz überwiegend wurde bisher der Nachweis eines medizinischen Nutzens durch randomisierte klinische Studien nachgewiesen. An den bisher durchgeführten und akzeptierten Studienergebnissen wird aber auch immer wieder Kritik geäußert, die teilweise berechtigt ist, teilweise aber auch systembedingt nicht korrigiert werden kann. So werden z. B. Verzerrungseffekte mit fehlender Verblindung, mögliche Placeboeffekte, hohe Drop-out-Raten und Prä-post-Vergleiche mit dem Risiko der Überschätzung der Therapieeffekte angeführt [12, 15, 19, 20, 22]. Diese Aspekte werden im Assessment des BfArM berücksichtigt und mit Antragstellern diskutiert, dazu gehören u. a. die Robustheit des Effektes anhand von Sensitivitätsanalysen. Darüber hinaus prüft das BfArM die Plausibilität und Konsistenz der Effekte des positiven Versorgungseffektes und beachtet sorgfältig methodische Aspekte (u. a. Verblindung, Ersetzung fehlender Werte, Randomisierung) in der Gesamtbeurteilung. Regelhaft seit Beginn des Verfahrens erfolgt eine konservative Ersetzung fehlender Werte mittels referenzbasierter Imputation (z. B. Jump-to-reference-Methode) oder Nonresponderimputation bei Responderanalysen.

## Übersicht über DiGAs bei psychischen Erkrankungen

In Übereinstimmung mit entsprechenden Therapieleitlinien sind DiGAs zur Überbrückung von Wartezeiten bis zum Beginn einer Therapie oder komplementär zu laufenden Therapien oder zum Boostern einer stattgefundenen Therapie bei psychiatrischen Indikationen einzusetzen und können so eine niedrigschwellige Versorgungsalternative gegenüber persönlicher Therapie darstellen. Bei akuten Krankheitssituationen können sie nach Verordnung sofort angewendet werden und insbesondere bei der Wartezeit auf einen Therapieplatz eine wertvolle erste Hilfe darstellen.

**Abb. 4:**
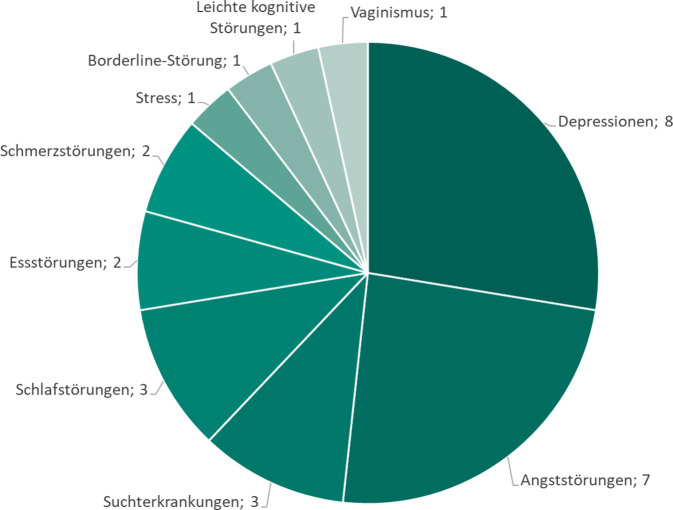
Übersicht zu Indikationsbereichen gelisteter digitaler Gesundheitsanwendungen im psychischen Bereich

Vor der Verordnung einer DiGA muss immer eine entsprechende Diagnostik stattgefunden haben. Achtundzwanzig von den 59 derzeit verschreibungsfähigen DiGAs befassen sich mit psychiatrischen Indikationen, v. a. im Bereich depressiver und Angststörungen (Abb. 4). Diese DiGAs bereiten mehr oder weniger Inhalte aus dem Bereich der kognitiven Verhaltenstherapie (KVT) auf. Auch in Anwendungen mit primär somatischer Indikation finden sich häufig psychotherapeutische Inhalte, wie beispielsweise in Anwendungen zu chronischen Schmerzen oder bei Brustkrebs [20]. DiGAs können als browserbasierte Webanwendungen oder als Smartphone- bzw. Tablet-Apps sowie als Kombinationen aus beiden eingesetzt werden. Viele DiGAs sind modular aufgebaut und bereiten umfangreich psychoedukative Inhalte auf, die das Störungsbild und psychotherapeutische Erklärungsmodelle, z. B. kognitive Verzerrungen bei Depression oder den Angstkreis bei Angststörungen, erklären. Oft werden diese sehr anschaulich multimedial ergänzt mit z. B. Videos oder Audiodateien, anhand von Tagebuchfunktionen und Symptomchecklisten lassen sich Symptomausprägung und Aktivitäten im Verlauf beurteilen. Dies wird kombiniert und ergänzt mit konkreten auf das Störungsbild abgestimmten Übungen, z. B. mit Fragebögen, die mit Auslösesituationen assoziierte automatische Gedanken und Gefühle bewusst und alternative, funktionalere Gedanken möglich machen sollen. Entspannungsverfahren können ebenso über digitale Interventionen erlernt und geübt werden. Einige der DiGAs bieten über Chat- und Mailfunktionen die Möglichkeit, individuelles Feedback zu erhalten, Rückfragen zu stellen oder Kriseninterventionen zu ermöglichen. Bei all diesen DiGAs finden sich Empfehlungen, wie häufig sie minimal pro Woche eingesetzt werden sollen, manche haben dafür spezielle Erinnerungs- und Motivationsfunktionen. Es lohnt sich also, eine DiGA je nach den in ihr gesetzten Schwerpunkten und Feedbacktechniken mit dem Patienten individuell auszusuchen [1, 11, 13, 18, 22]. DiGAs bieten zudem die Möglichkeit einer Verzahnung von digitalen Therapieanteilen mit einem persönlichen Arzt-Patienten-Kontakt (sog. Blended-care-Ansatz; [2]). Diese Ansätze werden seit einiger Zeit auch für andere Erkrankungen gefordert [23]. Das Behandlungsprogramm kann seitens des Therapeuten für den Patienten individuell abgestimmt werden. So können in der Zeit zwischen Psychotherapiesitzungen Inhalte in der App vertieft, gefestigt oder ergänzt werden [9, 24].

## Was ist bei der Verordnung von DIGAs zu beachten?

Vor der Verordnung ist eine Diagnosestellung unter Beachtung aller absoluten und relativen Kontraindikationen sowie weiteren Ausschlusskriterien notwendig. Dem DiGA-Verzeichnis lässt sich zudem eine Beschreibung der seitens der DiGA-Hersteller zugeschriebenen Rolle der verordnenden Ärzte und Psychotherapeuten entnehmen, eine Verordnung ist aber auch aus der stationären Behandlung über das Entlassmanagement möglich. Die Verordnung gilt in der Regel für 90 Tage, Folgeverordnungen derselben DiGA sowie die Verordnung mehrerer DiGAs an Patienten sind grundsätzlich möglich, solange dies therapeutisch sinnvoll erscheint und mit dem Wirtschaftlichkeitsprinzip vereinbar bleibt, z. B. um Booster-Effekte oder verbesserte Transferleistungen in den individuellen Alltag zu erzielen. Um sich einen generellen Überblick über die Inhalte der jeweiligen DiGA zu verschaffen, bieten Hersteller häufig kostenlose Testversionen an. Darüber stehen verschiedene Kontaktmöglichkeiten für Anwender und Therapeuten bei technischen Problemen oder inhaltlichen Fragen zu den DiGAs per Telefon- oder Mail-Hotline zur Verfügung. Bei einigen DiGAs besteht die Notwendigkeit zusätzlicher, begleitender Leistungen durch die behandelnden Ärzte und Psychotherapeuten, entsprechende vertragsärztliche Tätigkeiten werden im DiGA-Verzeichnis separat ausgewiesen und können entsprechend vergütet werden. Ganz überwiegend werden DiGAs von Ärzten und Psychotherapeuten verordnet, nur ca. 10 % werden direkt bei der Krankenkasse beantragt.

**Abb. 5:**
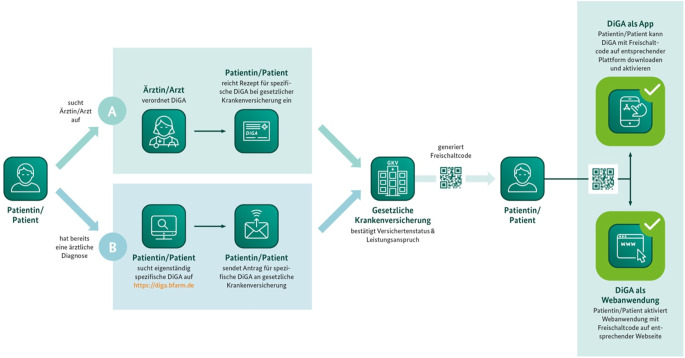
Möglichkeiten zur Verordnung und Erstattungsweg einer digitalen Gesundheitsanwendung (*DiGA*)

Nach Einreichung des Rezepts bei ihrer gesetzlichen Krankenkasse erhalten Versicherte einen 16-stelligen Freischaltcode, mit dem sie die Anwendung als App oder Webanwendung herunterladen bzw. freischalten können (Abb. 5), im Laufe des Jahres 2025 soll es hierfür auch eine elektronische Einreichungsmöglichkeit sowie eine Frist von 2 Arbeitstagen für den Erhalt des Freischaltcodes geben [9]. Ein wichtiger Aspekt aus Patientensicht ist, dass diese bislang zu DiGA-Verordnungen keine Zuzahlung leisten müssen. Privatversicherte müssen die Erstattungsfähigkeit mit ihrer Kasse vorab klären, in der Regel wird dem aber stattgegeben.

## Fazit für die Praxis

- Digitale Gesundheitsanwendungen (DiGAs) sind ein wichtiger Baustein in der digitalen Gesundheitsversorgung. Durch niedrigschwellige Anwendung und geringe oder nicht vorhandene Nebenwirkungen haben DiGAs das Potenzial, die Verteilungsgerechtigkeit z. B. in ländlichen Gebieten mit wirksamen Therapieoptionen zu stärken.
- Durch eine mehrheitlich ärztliche Verordnung, Blended-care- und Telemonitoringansätze, schrittweise Unterstützung der elektronischen Patientenakte und notwendige, begleitende vertragsärztliche Tätigkeiten mit teilweise Einstellmöglichkeiten durch professionelle Anwender haben DiGAs das Ziel, durch ihren gezielten Einsatz komplementär die ärztliche und psychotherapeutische Versorgung effizienter und wirksamer zu gestalten.
- Basierend auf den bisher gemachten Erfahrungen aktualisiert das BfArM seinen Leitfaden für Hersteller und führt den Dialog mit Gesetzgeber, Herstellern, Verordnern, Nutzern und Kostenträgern fort.

## Supplementary Information

Infobox: Wichtige Begriffe in der digitalen Gesundheitsversorgung im Bereich Psychiatrie und Psychotherapie
